# Supplementary material for: Phylogenomic analysis of Clostridioides difficile ribotype 106 strains reveals novel genetic islands and emergent phenotypes
Source: Sci Rep. 2020 Dec 17;10:22135. doi: 10.1038/s41598-020-79123-2 (PMC7747571; doi:10.1038/s41598-020-79123-2)
Supplement: Supplementary file 1 — Supplementary Information 1. [file 41598_2020_79123_MOESM1_ESM.docx]

**Phylogenomic analysis of *Clostridioides difficile* ribotype 106 strains reveals novel genetic islands and emergent phenotypes**

Bryan Angelo P. Roxas^1,^ ^†^, Jennifer Lising Roxas^1, †^, Rachel Claus-Walker^1^, Anusha Harishankar^1^, Asad Mansoor^1^, Farhan Anwar^1^, Shobitha Jillella^1^, Alison Williams^1^, Jason Lindsey^1^, Sean P. Elliott^2^, Kareem W. Shehab^2^, V.K. Viswanathan^1,3,4^

and Gayatri Vedantam^1,3,4,5^

^1^ School of Animal and Comparative Biomedical Sciences, The University of Arizona, Tucson, AZ, United States

^2^ De­partment of Pediatrics, The University of Arizona College of Medicine, Tucson, AZ, United States

^3^ Department of Immunobiology, The University of Arizona, Tucson, AZ, United States

^4^ Bio5 Institute for Collaborative Research, The University of Arizona, Tucson, AZ, United States

^5^ Southern Arizona VA Health Care System, Tucson, AZ, United States

^†^ Bryan Angelo P. Roxas and Jennifer Lising Roxas contributed equally to this work.

Correspondence:

Gayatri Vedantam

School of Animal and Comparative Biomedical Sciences

University of Arizona

1117 E Lowell St, Bldg. 90, Room 227

Tucson, AZ 85721

[gayatri@email.arizona.edu](mailto:gayatri@email.arizona.edu)

**SUPPLEMENTARY MATERIALS**

**SUPPLEMENTAL METHODS**

***C. difficile infection of Golden Syrian hamsters.***  A pilot hamster study, approved by the Institutional Animal Care and Use Committee of the University of Arizona, was conducted to test GV599 virulence. Six-week old male Golden Syrian hamsters (weighing 90-110 grams) from Charles River Laboratory (Wilmington, MA) were given antibiotics prior to *C. difficile* infection. A single oral dose of clindamycin (30 mg/kg) was administered three days prior to infection (Day -3), while chloramphenicol (50 mg/kg) was administered orally for a total of 6 doses prior to infection (2 doses on Day -3, 3 doses on Day -2, and 1 dose on Day -1). Three hamsters were infected with GV599 (164 spores; orally administered in PBS). The control hamster was given both antibiotics and PBS. Animals were monitored for disease symptoms (wet tail, ruffled coat, lethargy, weight loss). Moribund hamsters or those meeting the criteria for euthanasia were administered 270 mg/kg Euthanasia III (MedPharma Inc, Pomona, CA, United States). Euthanized hamsters were dissected for visualization of gross pathology, and cecal contents harvested and plated on selective TCCFA to confirm *C. difficile* colonization. Colonies recovered were ribotyped to confirm RT106 infection. Cecal tissue samples were fixed with 10% neutral buffered formalin and submitted to Arizona Veterinary Diagnostic Laboratory (Tucson, AZ) for hematoxylin and eosin staining.

**SUPPLEMENTAL DATA**

­­­
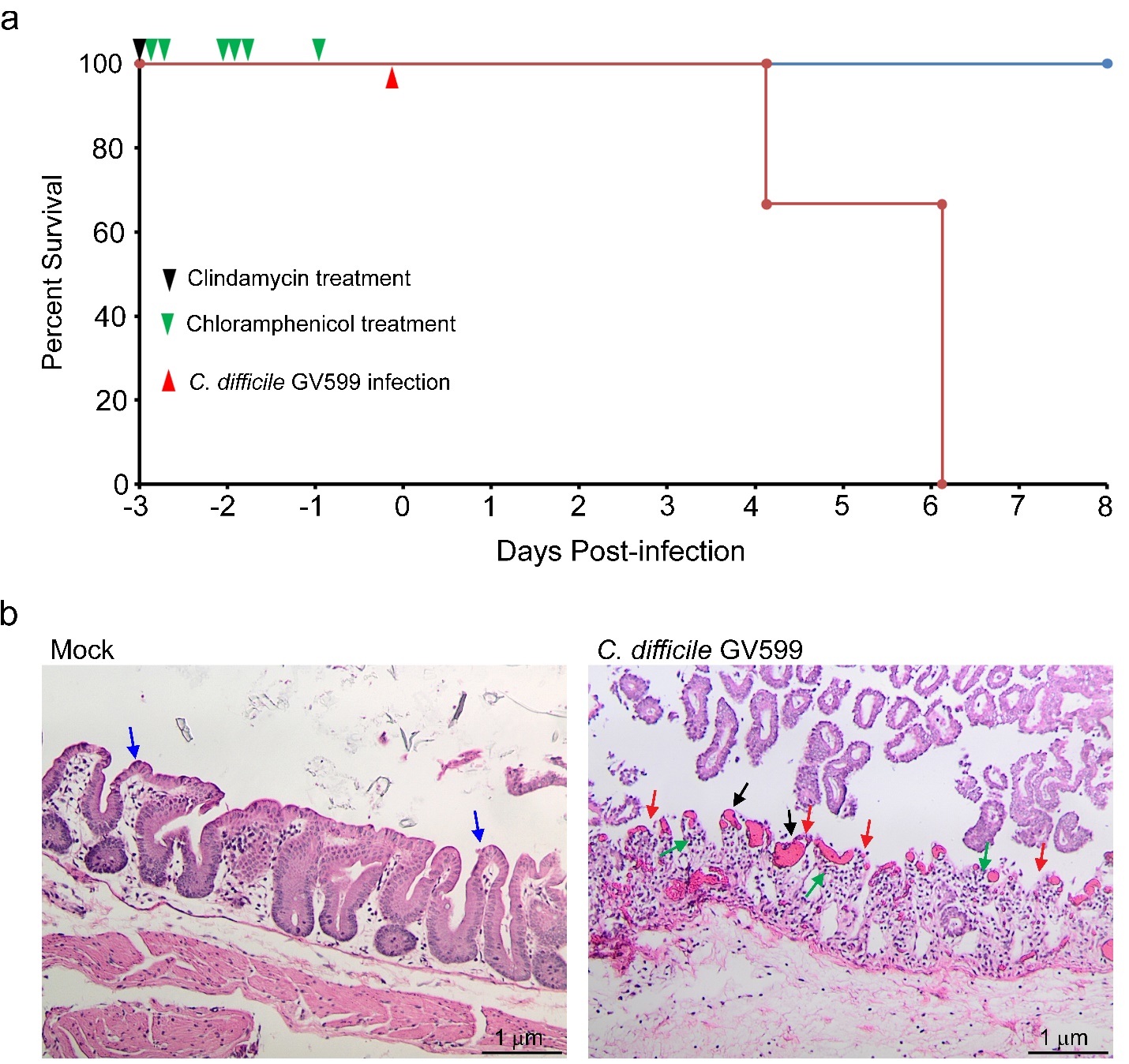


**Supplemental Figure S1. Golden Syrian hamsters succumbed to *C. difficile* GV599 infection. a**, Infection of Golden Syrian hamsters with GV599 (164 spores in PBS; administered orally) caused 100% mortality (red line; n=3). **b**, Microscopic images of hematoxylin-eosin-stained colonic tissues of GV599-infected hamsters revealed classic *C. difficile* infection pathology including gross hemorrhage (black arrows), epithelial erosion (red arrow) and recruitment of inflammatory infiltrates (green arrows). In comparison, colonic tissue sections of the mock-treated hamster had intact intestinal epithelium (blue arrow). Images shown are representative of 27 fields of view per animal.

­
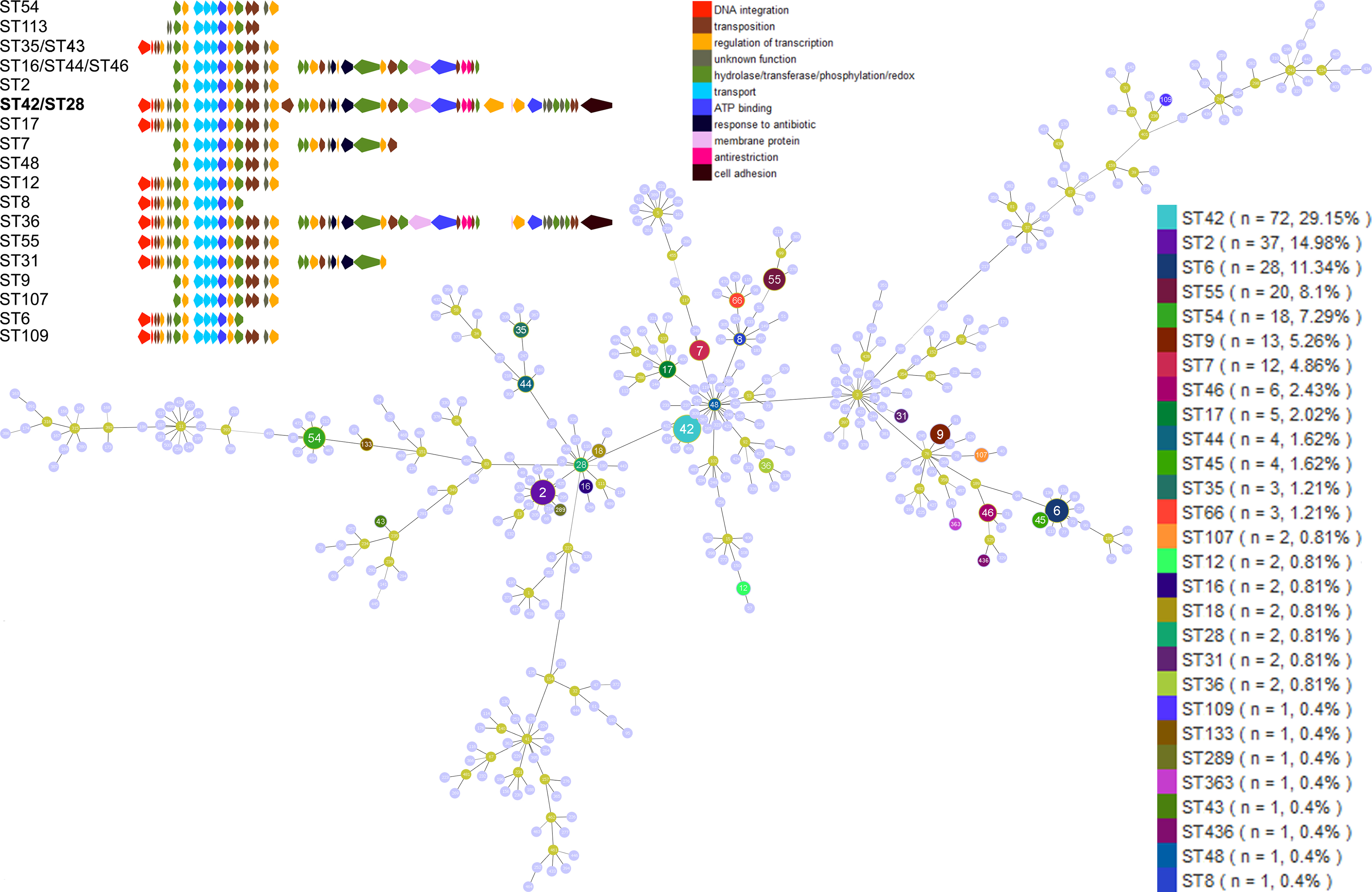


**Supplemental Figure S2. Sequence type does not recapitulate carriage of the 46 kb island 1.** Minimum spanning tree (MST) of the *C. difficile* sequence types (ST) that carry the whole or part of the 46 kb genomic island 1. Analysis was performed on MLST allele data from PubMLST (<https://pubmlst.org/>). Tree shows relatedness of the STs independent of genomic island 1.

**Supplemental Table S1**. Antibiotic resistance genes present in the RT106 strains

| Antimicrobial Resistance | Antibiotic | macrolide-lincosamide-streptogramin B group | | | Fluoroquinolone | | | | | Teicoplanin | | |
| --- | --- | --- | --- | --- | --- | --- | --- | --- | --- | --- | --- | --- |
|  | Gene Family | ErmB | ErmB | 23S rRNA | GyrA | | | | GyrB | VanZA | | VanZA |
|  | Locus ID | CD630_20070 | CD630_20100 | FE556_13790 | FE556_18445 | | | | FE556_18440 | FE556_05915 | | FE556_11215 |
|  | Gene | *ermB1* | *ermB* | *rrn* | *gyrA* | | | | *gyrB* | *vanZ1* | | *vanZ* |
|  | Mutations | - | - | 656C>T | D71V, D81N, T82I, T82V, or A118T | L406I | A421T | D468N | D426V, D426N, R447L, R447K, S366A or S416A | I46V | A99V | - |
| Strains | GV371 | x | x | x | x | x | x | x | x | x | x | **** |
|  | GV423 | x | x | x | x | x | x | x | x | x | x | **** |
|  | GV432 | x | x | x | x | x | x | x | x | x | x | **** |
|  | GV597 | x | x | x | x | x | **** | x | x | x | x | **** |
|  | GV453 | x | x | x | x | x | x | x | x | x | x | **** |
|  | GV587 | x | x | x | x | x | x | x | x | x | x | **** |
|  | GV642 | x | x | x | x | x | x | x | x | x | x | **** |
|  | GV364 | x | x | x | x | x | x | x | x | x | x | **** |
|  | GV375 | x | x | x | x | x | x | x | x | x | x | **** |
|  | GV377 | x | x | x | x | x | x | x | x | x | x | **** |
|  | GV415 | x | x | **** | x | x | x | x | x | x | x | **** |
|  | GV421 | x | x | x | x | x | x | x | x | x | x | **** |
|  | GV425 | x | x | x | x | x | x | x | x | x | x | **** |
|  | GV426 | x | x | x | x | x | x | x | x | x | x | **** |
|  | GV524 | x | x | x | x | x | x | x | x | x | x | **** |
|  | GV576 | x | x | x | x | x | x | x | x | x | x | **** |
|  | GV589 | x | x | x | x | x | x | x | x | x | x | **** |
|  | GV753 | x | x | x | x | x | x | x | x | x | x | **** |
|  | GV599 | x | x | x | x | x | x | x | x | x | x | **** |
|  | GV457 | x | x | x | x | x | x | x | x | x | x | **** |
|  | GV515 | x | x | x | x | x | x | x | x | x | x | **** |
|  | 630 | **** | **** | x | x | x | x | x | x | x | x | x |
|  | VPI | x | x | x | x | x | x | x | x | x | x | x |
|  | BI-1 | x | x | x | x | **** | x | **** | x | **** | **** | x |
| Notes:** **  **  **denotes presence of gene and/or mutation associated with antibiotic resistancePage | | | | | | | | | | | | |
| x denotes absence of gene and/or mutation associated with antibiotic resistance | | | | | | | | | | | | |

**
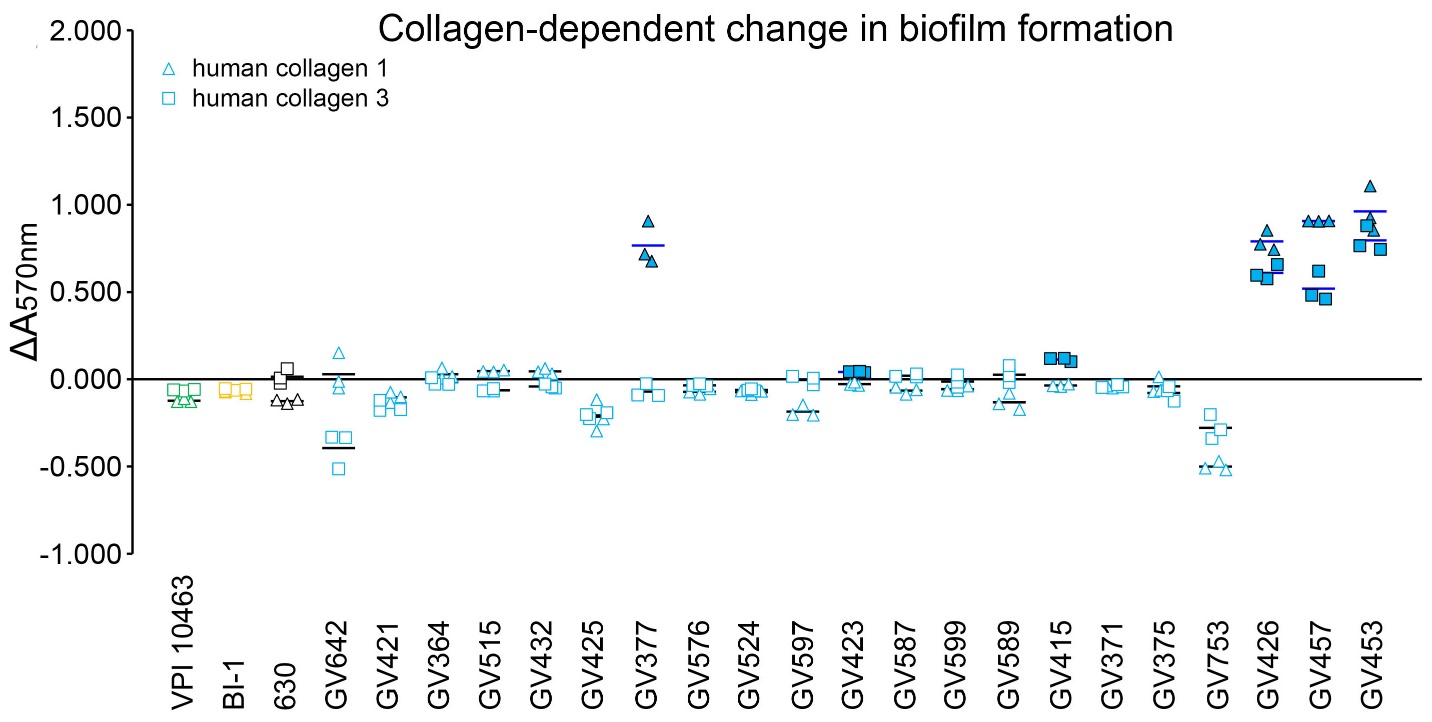
Supplemental Figure S3. Clinical RT106 isolates form dense biofilms on human type I and type III collagen.** 21 clinical RT106 strains (blue) and 3 non-RT106 toxigenic *C. difficile* strains (VPI, BI-1, and 630 designated as green, yellow and black, respectively) were cultured for 72 hours in uncoated wells or wells coated with human type I or type III collagen. Relative changes in biofilm densities (ΔA_570nm_) were determined by comparing A_570nm_ of crystal violet-stained biofilms formed on human type I or type III collagen vs. on uncoated plastic wells. Triangles denote ΔA_570nm_ for human type I collagen, while squares denote ΔA_570nm_ for human type III collagen. Filled blue triangles or squares denote P_value_ < 0.05 determined using Student’s t test to compare mean A_570nm_ by each strain on collagen-coated vs. uncoated wells. No difference in biofilm formation was observed when the reference *C. difficile* 630, BI-1 and VPI strains were cultured on wells with or without collagen. RT106 strains displayed denser biofilms on collagen-coated wells (One-sample one-tailed T-test; H_alt_: mean ΔA_570nm_>0; H_0_: mean ΔA_570nm_=0; P_value_=0.13903 for type I collagen data set; P_value_=0.305584 for type III collagen data set).


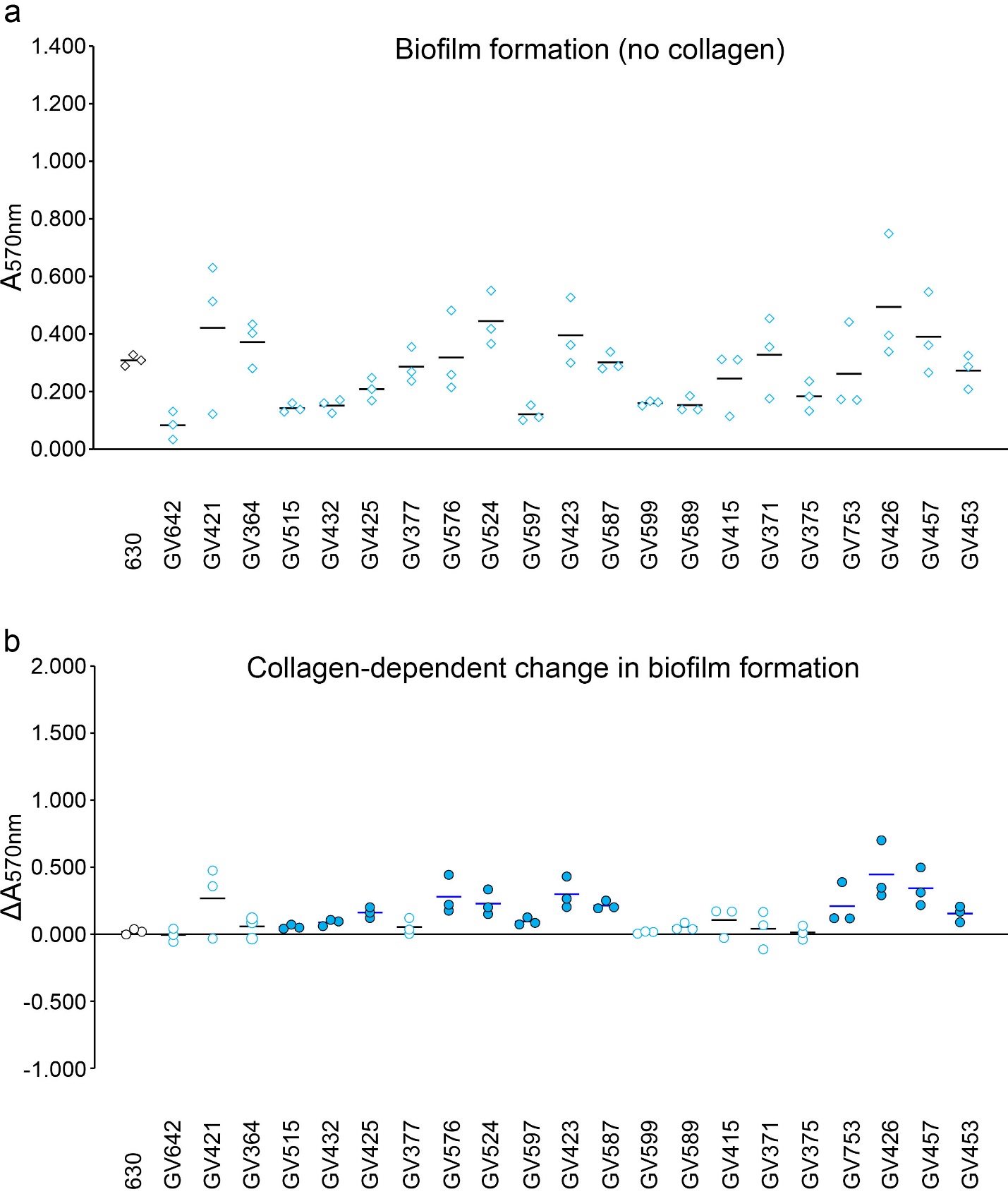


**Supplemental Figure S4. Clinical RT106 isolates form dense biofilms on rat tail type I collagen. a**, 21 clinical RT106 strains (blue) and a non-RT106 toxigenic *C. difficile* 630 strain (black) were cultured for 72 hours in uncoated wells or wells coated with human type I or type III collagen. RT106 isolates displayed variable biofilm densities on abiotic plastic wells. **b,** Relative changes in biofilm densities (ΔA_570nm_) were determined by comparing A_570nm_ of crystal violet-stained biofilms formed on rat tail type I collagen vs. on uncoated plastic wells. Filled blue circles denote P_value_ < 0.05 determined using Student’s t test to compare mean A_570nm_ by each strain on collagen-coated vs. uncoated wells. No difference in biofilm formation was observed when the reference *C. difficile* 630 was cultured on wells with or without collagen. Overall, RT106 strains displayed denser biofilms on collagen-coated wells (One-sample one-tailed T-test; H_alt_: mean ΔA_570nm_>0; H_0_: mean ΔA_570nm_=0; P_value_=0.00001).

**Supplemental Table S2.** List of RT106 Strains

| **Strain** | **Isolation Year** | **Clinical Test (PCR or GDH/EIA Result)** | **Genome Size (bases)** | **GC Content (%)** | **Phenotypic Characterization Performed? (Yes or No)** |
| --- | --- | --- | --- | --- | --- |
| GV364 | 2015 | PCR: + | 4043408 | 28.4 | Yes |
| GV371 | 2015 | PCR: + | 4062530 | 28.4 | Yes |
| GV375 | 2016 | PCR: + | 4045723 | 28.4 | Yes |
| GV377 | 2016 | PCR: + | 4081010 | 28.5 | Yes |
| GV415 | 2016 | PCR: + | 4052544 | 28.4 | Yes |
| GV421 | 2016 | PCR: + | 4012174 | 28.3 | Yes |
| GV423 | 2016 | PCR: + | 4050785 | 28.4 | Yes |
| GV425 | 2016 | PCR: + | 4014240 | 28.3 | Yes |
| GV426 | 2016 | PCR: + | 4013299 | 28.3 | Yes |
| GV432 | 2016 | PCR: + | 4051197 | 28.5 | Yes |
| GV453 | 2016 | PCR: + | 4126224 | 28.5 | Yes |
| GV457 | 2016 | PCR: + | 4067987 | 28.4 | Yes |
| GV515 | 2017 | GDH/EIA: +/+ | 4030448 | 28.3 | Yes |
| GV524 | 2017 | GDH/EIA: -/- | 4022675 | 28.3 | Yes |
| GV576 | 2017 | GDH/EIA: +/- | 4027239 | 28.3 | Yes |
| GV587 | 2017 | GDH/EIA: +/- | 4051442 | 28.4 | Yes |
| GV589 | 2017 | GDH/EIA: +/+ | 4061628 | 28.4 | Yes |
| GV597 | 2017 | GDH/EIA: +/- | 4059478 | 28.4 | Yes |
| GV599 | 2017 | GDH/EIA: +/+ | 4083315 | 28.5 | Yes |
| GV642 | 2017 | GDH/EIA: +/- | 4103497 | 28.4 | Yes |
| GV753 | 2017 | GDH/EIA: +/+ | 4094464 | 28.4 | Yes |
| GV814 | 2017 | GDH/EIA: +/+ | 4066154 | 28.5 | No |
| GV831 | 2017 | GDH/EIA: +/+ | 4048397 | 28.5 | No |
| GV836 | 2017 | GDH/EIA: +/+ | 4039872 | 28.4 | No |
| GV840 | 2017 | GDH/EIA: +/+ | 4066224 | 28.4 | No |
| GV868 | 2018 | GDH/EIA: +/+ | 4026007 | 28.4 | No |
| GV870 | 2018 | GDH/EIA: +/+ | 4080981 | 28.8 | No |
| GV962 | 2018 | GDH/EIA: +/+ | 4086548 | 28.4 | No |
| GV973 | 2018 | GDH/EIA: +/+ | 4112429 | 28.6 | No |
| GV986 | 2018 | GDH/EIA: +/+ | 4037148 | 28.4 | No |
| GV996 | 2018 | GDH/EIA: +/+ | 4112462 | 28.7 | No |
| GV997 | 2018 | GDH/EIA: +/+ | 4053485 | 28.5 | No |
| GV1002 | 2018 | GDH/EIA: +/+ | 4097153 | 29.0 | No |
| GV1006 | 2018 | GDH/EIA: +/+ | 4412809 | 29.2 | No |
| GV1057 | 2018 | GDH/EIA: +/+ | 4038504 | 28.5 | No |
| GV1105 | 2018 | GDH/EIA: +/+ | 4070520 | 28.7 | No |
| GV1125 | 2018 | GDH/EIA: +/+ | 4028672 | 28.4 | No |
| GV1152 | 2018 | GDH/EIA: +/+ | 4055778 | 28.4 | No |

| **Locus ID** | **Product** | **Start** | **End** | **Strand** |
| --- | --- | --- | --- | --- |
| FE556_11090 | site-specific integrase | 201569 | 202759 | - |
| FE556_11095 | excisionase | 202837 | 203040 | - |
| FE556_11100 | hypothetical protein | 203145 | 203382 | + |
| FE556_11105 | helix-turn-helix domain-containing protein | 203412 | 203666 | - |
| FE556_11110 | sigma-70 family RNA polymerase sigma factor | 203669 | 204088 | - |
| FE556_11115 | hypothetical protein | 204355 | 204585 | - |
| FE556_11120 | DUF4177 domain-containing protein | 204609 | 204800 | - |
| FE556_11125 | class I SAM-dependent methyltransferase | 204964 | 205680 | - |
| FE556_11130 | TetR/AcrR family transcriptional regulator | 205802 | 206428 | - |
| FE556_11135 | cation transporter | 206910 | 207821 | - |
| FE556_11140 | ABC transporter permease | 207833 | 208531 | - |
| FE556_11145 | ABC transporter permease | 208528 | 209211 | - |
| FE556_11150 | ABC transporter ATP-binding protein | 209208 | 210053 | - |
| FE556_11155 | TetR/AcrR family transcriptional regulator | 210157 | 210744 | - |
| FE556_11160 | alpha/beta hydrolase | 210816 | 211673 | - |
| FE556_11165 | conjugal transfer protein | 211886 | 212497 | - |
| FE556_11170 | bifunctional lytic transglycosylase/NlpC/P60 family protein | 212448 | 213188 | - |
| FE556_11175 | DUF3788 domain-containing protein | 213656 | 214090 | - |
| FE556_11180 | helix-turn-helix domain-containing protein | 214168 | 215079 | - |
| FE556_11185 | IS110 family transposase | 215358 | 216527 | + |
| FE556_11190 | GNAT family N-acetyltransferase | 216917 | 217468 | - |
| FE556_11195 | SAM-dependent methyltransferase | 217482 | 217958 | - |
| FE556_11200 | MerR family transcriptional regulator | 218039 | 218854 | - |
| FE556_11205 | conjugal transfer protein | 218957 | 219529 | - |
| FE556_11210 | DUF4865 family protein | 219799 | 220074 | - |
| FE556_11215 | VanZ family protein | 220079 | 220585 | - |
| FE556_11220 | XRE family transcriptional regulator | 220688 | 220881 | + |
| FE556_11225 | tetracycline resistance MFS efflux pump | 221068 | 222231 | - |
| FE556_11230 | pyruvate, phosphate dikinase | 222221 | 224788 | - |
| FE556_11235 | TetR/AcrR family transcriptional regulator | 224785 | 225396 | - |
| FE556_11240 | conjugal transfer protein | 225540 | 226442 | - |
| FE556_11245 | bifunctional lytic transglycosylase/NlpC/P60 family protein | 226462 | 227466 | - |
| FE556_11250 | MFS transporter | 227463 | 229658 | - |
| FE556_11255 | ATP-binding protein | 229658 | 232108 | - |
| FE556_11260 | conjugal transfer protein | 232086 | 232484 | - |
| FE556_11265 | antirestriction protein ArdA | 232605 | 233108 | - |
| FE556_11270 | antirestriction protein ArdA | 233126 | 233629 | - |
| FE556_11275 | conjugal transfer protein | 233548 | 233844 | - |
| FE556_11280 | GNAT family N-acetyltransferase | 233951 | 234331 | - |
| FE556_11285 | hypothetical protein | 234409 | 234570 | - |
| FE556_11290 | group II intron reverse transcriptase/maturase ltrA | 234737 | 236647 | - |
| FE556_11295 | DUF3789 domain-containing protein | 237389 | 237523 | - |
| FE556_11300 | replication initiation factor domain-containing protein | 237530 | 238651 | - |
| FE556_11305 | ATP-binding protein | 238931 | 240325 | - |
| FE556_11310 | YcxB family protein | 240436 | 240747 | - |
| FE556_11315 | DUF3795 domain-containing protein | 240775 | 241317 | - |
| FE556_11320 | alpha/beta hydrolase | 241378 | 241980 | - |
| FE556_11325 | DUF3788 domain-containing protein | 242035 | 242448 | - |
| FE556_11330 | glyoxalase | 242512 | 242970 | - |
| FE556_11335 | DUF961 domain-containing protein | 243051 | 243470 | - |
| FE556_11340 | DUF961 domain-containing protein | 243479 | 243802 | - |
| FE556_11345 | hypothetical protein | 243759 | 244011 | - |
| FE556_11350 | SrtB-anchored collagen-binding adhesin | 244024 | 247071 | - |

**Supplemental Table S3. List of genes within genomic island 1**

**Supplemental Table S4. List of genes within genomic island 3**

| **Locus ID** | **Product** | **Start** | **Stop** | **Strand** |
| --- | --- | --- | --- | --- |
| FE556_02390 | SrtB-anchored collagen-binding adhesin | 145981 | 149034 | + |
| FE556_02395 | DNA cytosine methyltransferase | 149035 | 150111 | + |
| FE556_02400 | DUF961 domain-containing protein | 150312 | 150635 | + |
| FE556_02405 | DUF961 domain-containing protein | 150652 | 151020 | + |
| FE556_02410 | ATP-binding protein | 151038 | 152435 | + |
| FE556_02415 | XRE family transcriptional regulator | 152703 | 153929 | + |
| FE556_02420 | DUF3789 domain-containing protein | 153913 | 154056 | + |
| FE556_02425 | hypothetical protein | 154057 | 154278 | + |
| FE556_02430 | iron-sulfur protein | 154352 | 155113 | + |
| FE556_02435 | conjugal transfer protein | 155212 | 155433 | + |
| FE556_02440 | antirestriction protein ArdA | 155430 | 155915 | + |
| FE556_02445 | antirestriction protein ArdA | 155932 | 156435 | + |
| FE556_02450 | conjugal transfer protein | 156521 | 156910 | + |
| FE556_02455 | ATP-binding protein | 156897 | 159347 | + |
| FE556_02460 | YtxH domain-containing protein | 159410 | 161512 | + |
| FE556_02465 | peptidase P60 | 161509 | 162519 | + |
| FE556_02470 | conjugal transfer protein | 162537 | 163448 | + |
| FE556_02475 | TetR/AcrR family transcriptional regulator | 163589 | 164215 | + |
| FE556_02480 | ABC transporter ATP-binding protein | 164212 | 165099 | + |
| FE556_02485 | ABC transporter permease | 165096 | 165893 | + |
| FE556_02490 | ABC transporter ATP-binding protein | 166073 | 166759 | + |
| FE556_02495 | ABC transporter permease | 166749 | 169307 | + |
| FE556_02500 | response regulator transcription factor | 169372 | 170037 | + |
| FE556_02505 | HAMP domain-containing histidine kinase | 170040 | 171065 | + |
| FE556_02510 | sigma-70 family RNA polymerase sigma factor | 171349 | 171771 | + |
| FE556_02515 | helix-turn-helix domain-containing protein | 171776 | 172015 | + |
| FE556_02520 | phosphoesterase | 172468 | 173319 | + |
| FE556_02525 | DUF4368 domain-containing protein | 173612 | 175225 | + |

**Supplemental Table S5. Increase in minimum inhibitory concentration after sub-inhibitory exposure to teicoplanin**

| **Strain** | **Fold change in teicoplanin resistance**  **post-induction** |
| --- | --- |
| GV415 | + 1.33 |
| GV425 | +2.00 |
| GV432 | +1.33 |
| GV457 | +1.52 |
| GV576 | +1.47 |
| GV589 | +1.47 |
| GV753 | +4.04 |

**Supplemental Table S6. GenBank Accession numbers of RT106 isolates**

| **Strain** | **NCBI Accession Numbers** | | | **Coverage** |
| --- | --- | --- | --- | --- |
|  | **Biosample** | **BioProject** | **WGS** |  |
| GV364 | SAMN11637479 | PRJNA542726 | VCAN00000000 | 608x |
| GV371 | SAMN11637480 | PRJNA542726 | VCAM00000000 | 463x |
| GV375 | SAMN11637481 | PRJNA542726 | VCAL00000000 | 543x |
| GV377 | SAMN11637482 | PRJNA542726 | VCAK00000000 | 550x |
| GV415 | SAMN11637483 | PRJNA542726 | VCAJ00000000 | 383x |
| GV421 | SAMN11637484 | PRJNA542726 | VCAI00000000 | 476x |
| GV423 | SAMN11637485 | PRJNA542726 | VCAH00000000 | 414x |
| GV425 | SAMN11637486 | PRJNA542726 | VCAG00000000 | 436x |
| GV426 | SAMN11637487 | PRJNA542726 | VCAF00000000 | 594x |
| GV432 | SAMN11637488 | PRJNA542726 | VCAE00000000 | 512x |
| GV453 | SAMN11637490 | PRJNA542726 | VCAC00000000 | 163x |
| GV457 | SAMN11637489 | PRJNA542726 | VCAD00000000 | 146x |
| GV515 | SAMN11637491 | PRJNA542726 | VCAB00000000 | 161x |
| GV524 | SAMN11637492 | PRJNA542726 | VCAA00000000 | 89x |
| GV576 | SAMN11637493 | PRJNA542726 | VBZZ00000000 | 101x |
| GV587 | SAMN11637496 | PRJNA542726 | VBZW00000000 | 110x |
| GV589 | SAMN11637497 | PRJNA542726 | VBZV00000000 | 78x |
| GV597 | SAMN11637495 | PRJNA542726 | VBZX00000000 | 117x |
| GV599 | SAMN11637494 | PRJNA542726 | VBZY00000000 | 114x |
| GV642 | SAMN11637498 | PRJNA542726 | VBZU00000000 | 91x |
| GV753 | SAMN11637499 | PRJNA542726 | VBZT00000000 | 126x |
| GV814 | SAMN11637501 | PRJNA542726 | VBZR00000000 | 30x |
| GV831 | SAMN11637500 | PRJNA542726 | VBZS00000000 | 28x |
| GV836 | SAMN11637502 | PRJNA542726 | VBZQ00000000 | 32x |
| GV840 | SAMN11637503 | PRJNA542726 | VBZP00000000 | 32x |
| GV868 | SAMN11637504 | PRJNA542726 | VBZO00000000 | 33x |
| GV870 | SAMN11637505 | PRJNA542726 | VCDT00000000 | 25x |
| GV962 | SAMN11637506 | PRJNA542726 | VBZN00000000 | 34x |
| GV973 | SAMN11637507 | PRJNA542726 | VBZM00000000 | 33x |
| GV986 | SAMN11637508 | PRJNA542726 | VBZL00000000 | 31x |
| GV996 | SAMN11637509 | PRJNA542726 | VBZK00000000 | 34x |
| GV997 | SAMN11637510 | PRJNA542726 | VBZJ00000000 | 22x |
| GV1002 | SAMN11637511 | PRJNA542726 | VBZI00000000 | 27x |
| GV1006 | SAMN11637512 | PRJNA542726 | VBZH00000000 | 30x |
| GV1057 | SAMN11637513 | PRJNA542726 | VBZG00000000 | 36x |
| GV1105 | SAMN11637514 | PRJNA542726 | VBZF00000000 | 17x |
| GV1125 | SAMN11637515 | PRJNA542726 | VBZE00000000 | 24x |
| GV1152 | SAMN11637516 | PRJNA542726 | VBZD00000000 | 35x |
