# Supplementary figures and images for: Phylogenomic analysis of Clostridioides difficile ribotype 106 strains reveals novel genetic islands and emergent phenotypes
Source: Sci Rep. 2020 Dec 17;10:22135. doi: 10.1038/s41598-020-79123-2 (PMC7747571; doi:10.1038/s41598-020-79123-2)

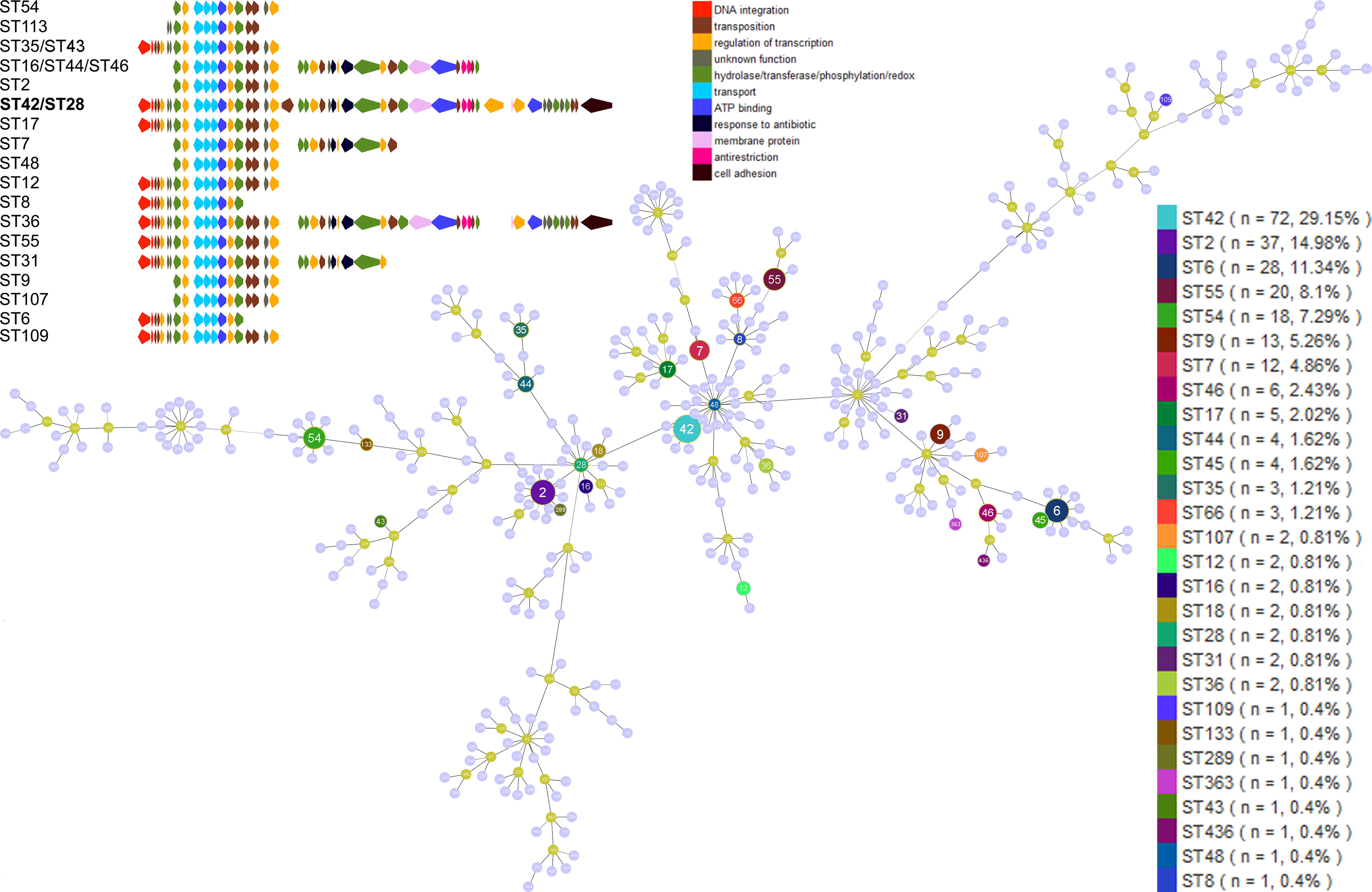

Supplement: Supplementary file 3 — Supplementary Figure S2. [file 41598_2020_79123_MOESM3_ESM.jpg]

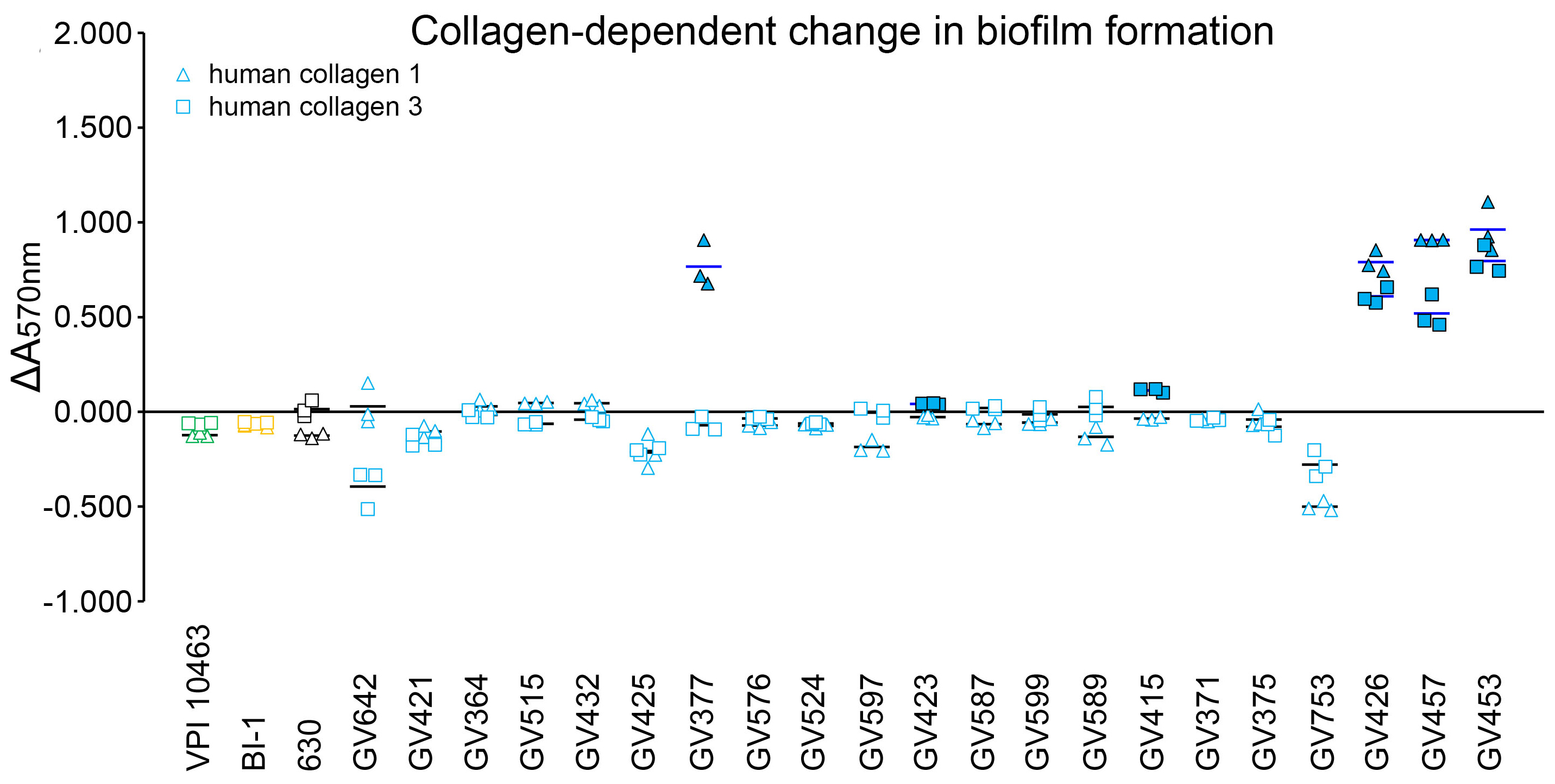

Supplement: Supplementary file 4 — Supplementary Figure S3. [file 41598_2020_79123_MOESM4_ESM.jpg]

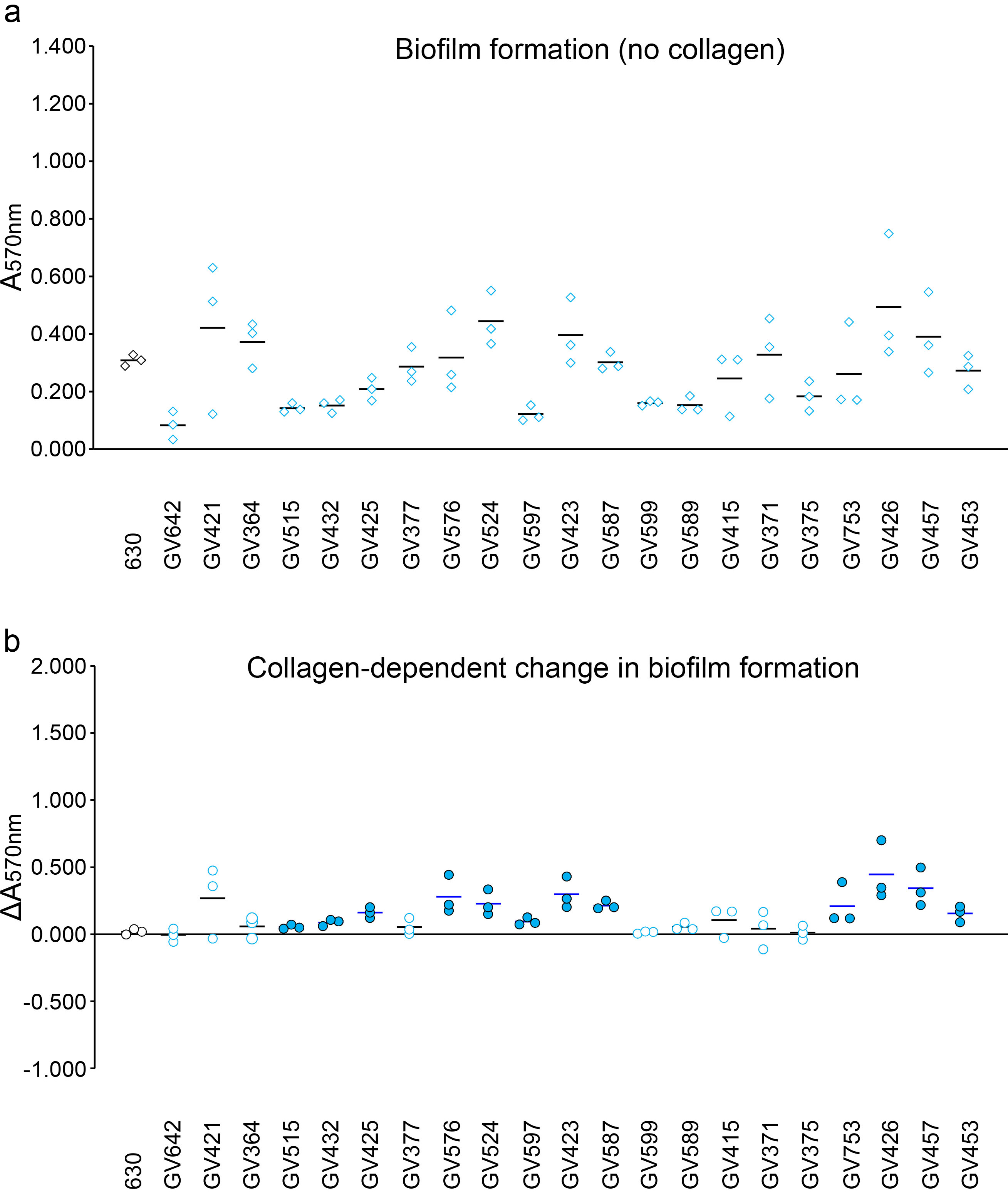

Supplement: Supplementary file 5 — Supplementary Figure S4. [file 41598_2020_79123_MOESM5_ESM.jpg]
